# Classification of Visual Smoothness Standards Using Multi-Scale Areal Texture Parameters and Low-Magnification Coherence Scanning Interferometry

**DOI:** 10.3390/ma17071653

**Published:** 2024-04-03

**Authors:** Jesse Redford, Brigid Mullany

**Affiliations:** Department of Mechanical Engineering, UNC Charlotte, Charlotte, NC 28223, USA

**Keywords:** visual appearance, powder coating, surface texture, multi-scale, orange peel, gloss, classification, machine learning

## Abstract

The ability to objectively specify surface finish to ensure consistent visual appearance addresses a vital need in surface coating engineering. This work demonstrates how a computational framework, called surface quality and inspection descriptors (*SQuID*™), can be leveraged to effectively rank different grades of surface finish appearances. ISO 25178-2 areal surface metrics extracted from bandpass-filtered measurements of a set of ten visual smoothness standards taken on a coherent scanning interferometer are used to quantify different grades of powder-coated surface finish. The ability to automatically classify the standard tiles using multi-scale areal texture parameters is compared to parameters obtained from a hand-held gloss meter. The results indicate that the ten different surface finishes can be automatically classified with accuracies as low as 65% and as high as 99%, depending on the filtering and parameters used to quantify the surfaces. The highest classification accuracy is achieved using only five multi-scale topography descriptions of the surface.

## 1. Introduction

The surface texture can impact a manufactured surface’s functionality and aesthetic quality. Some examples of where surface texture affects functional performance include wettability [1,2,3], reflectance [4,5], corrosion resistance [6,7], fatigue [7,8], and heat exchange [9,10]. While the surface aesthetic (visual and tactile) includes human perception and thus encroaches on the psychophysics domain [11,12,13,14], the topographical amplitudes and spatial relationships between different features on the surface, along with other traits such as color, thermal conductivity, and modulus, affect how the surface is perceived and valued [12,13,15,16,17,18]. Central to understanding how surface texture affects functionality and aesthetics is to isolate metrics capable of quantifying relevant surface topographies at the appropriate length scales. Similarly, quantitative metrics are needed to assist in fine-tuning processes used to generate the surfaces and for subsequent process control and part acceptance. That said, the identification of appropriate qualitative metrics is far from trivial [19,20]. While surfaces can be easily measured in digital environments, merely assigning a numerical assessment to a surface that does not correlate with its intended function provides incomplete and potentially misleading information. Additionally, while visual comparisons to defined standards are utilized in many instances, especially regarding aesthetics, human visual inspection (HVI) processes are inherently subjective, as discussed in [11]. There is a strong desire to replace HVI with automated systems and a longstanding goal of definitively linking functionality to surface topographies. However, there remains a need to make it easier to find suitable metrics that can reliably discriminate between surfaces grouped or ranked by functionality, subjective appearance, or, indeed, processing-induced differences [21,22,23].

### 1.1. Challenges in Quantifying Surface Quality

Information on surface quality can be obtained by multiple modes of acquisition and processing, giving different outputs. For example, functional gloss meter readings quantify a surface’s specular reflectance and/or the directional distribution of light reflected by surfaces illuminated under specified conditions [24]. The gloss unit (GU) reports the amount of luminous flux reflected from a specimen to that of a reference glass tile with a known refractive index (1.567) having a specular reflectance of 100 GU at a specified angle and wavelength (λ = 587.6 nm or 546.1 nm) [25]. Typical gloss meter systems are designed around standard measurement angles of 20° for high-gloss surfaces, 60° for semi-gloss surfaces, and 85° for matte surfaces [25,26]. Although multiple surface characteristics influence gloss values derived from these measurement angles, they may fail to characterize certain aspects that reduce appearance quality (haze, orange peel, etc.). As such, additional appearance terminology and parameters characterizing surface quality, such as the distinctness of image *DOI*, the “aspect of gloss characterized by the sharpness of images of objects produced by reflection at a surface”, have been developed [27] and used to quantify the severity of orange peel. *DOI* values close to 100 indicate a very high sharpness, and values close to 0 indicate a very high induced waviness in the reflected image [28]. Other industry standards have been developed by Rho-Point, two of which are *R_spec_* for smoothness quantification and the reflected image quality *RIQ* parameter [29]. On the other hand, conventional imaging techniques provide intensity images of how light interacts with the different spatial regions of the surface. While fast and effective in providing insights into the nature of the surface texture, they are sensitive to lighting conditions and lack topographical height data to provide full three-dimensional representations. Conventional image-processing metrics used to quantify surface textures, such as grayscale histograms, local binary patterns (LBP), and gray-level co-occurrence matrices (GLCMs), are often difficult to interpret with respect to the physical nature of the surface and thus have limited value in understanding what drives a particular functionality. As such, they will not be the focus of this paper.

A surface’s composition and geometric topography ultimately dictate its functional performance. Tactile or optical-based instruments can provide either line or areal height information pertaining to the geometric nature of the surface topography [30]. The speed at which a measurement is taken will vary depending on the system, with coherence scanning interferometry (CSI) being among the fastest but still slower than high-speed intensity imaging systems. Datasets from these systems can provide a wealth of geometrical topographical data with the scale of resolvable features dependent on system-limiting factors, tip radii for tactile measurements, and numerical aperture considerations for optical systems [31]. While the wealth of the available data is advantageous, effectively processing the data to give useful metrics still poses challenges [11,21]. Statistical parameters used to describe surfaces within the manufacturing community include height, spatial, hybrid, functional, and other parameters detailed in the ISO 25178-2 standard [32]. Even though there is a shift away from relying on two of the most commonly utilized areal metrics [33], the arithmetic average roughness, *Sa*, and the root mean square roughness, *Sq*, they remain prevalent despite having potentially similar values for surfaces that drastically differ in their topographical arrangements. Using other standardized parameters is promising, but questions about how alternative parameters should be selected for a specific application still arise. Surface geometries can be complicated; using only one or a small finite number of statistical parameters may fail to provide a full description. Increasing the number of parameters used can result in a more accurate description [34], but excessive reporting of redundant and/or irrelevant parameters leads to what Whitehouse coined in 1982 as “parameter rash” [35]. Further complicating the selection process, surface texture and its relationship to different functions is inherently multi-scale [22]. Single or multiple parameters calculated only at one specific spatial bandwidth may not always provide an adequate description. Even for the same surface, texture parameter values can change by orders of magnitude depending on the definition area of the measurement, sampling interval, form-removal operations, and cutoff frequencies used for high-, low-, or band-pass filtering of the data. If the search space is left unbounded, the number of parameter and length scale combinations are seemingly infinite. While particular combinations of areal parameters may provide a strong foundation for correlating process variations, function, or subjective visual appearance with statistical representations of the surface’s topography, it is crucial to understand that a heuristic approach to parameter selection might not always yield the most useful surface descriptions for a particular application. Objective and data-driven methodologies should complement heuristic approaches to ensure more accurate and effective surface characterizations, especially when aiming to capture subjective aspects of surface quality. However, using black-box machine learning (ML) models to carry out data-driven methodologies for classifying surface quality can limit the ability of designers, manufacturers, and researchers alike to use texture parameters to unambiguously communicate surface quality expectations and specifications for inspection. Therefore, achieving a balance between traditional methods and AI-driven approaches is essential. Integrating the strengths of both can lead to more robust and effective surface quality assessments while addressing the limitations of each approach.

### 1.2. Paper Description and Organization

To address the challenges of selecting the relevant length scale and parameters to reliably differentiate between different surface texture classes, this paper outlines an approach demonstrated on a set of ten ranked visual smoothness panels available from the Powder Coating Institute (PCI). These panels are commonly used in industry for HVI of powder coatings. Currently, there is no clear pathway for automatically classifying a workpiece finish as one of the ten PCI textured samples. The ASTM D3451-06 standard [36] for testing powder coatings notes that subjective HVI comparison is the most common method to quantify differences in the surface profile, primarily the levels of reflectance and orange peel of cured powder coatings. A second method refers to a portable instrument that, when scanned across the surface, acts like the human eye and detects differences in reflectance (light → dark areas), then transforms them into a numerical number relating to orange peel. A third method, alluded to within the standard, mentions a combination of surface amplitude and spatial content but clearly defines neither within.

To circumvent the need for subjective visual analysis, a previously published systematic framework, *SQuID*™ [21], will be built upon to derive quantitative descriptions using areal maps subdivided into multiple length scale measurements and a set of logical rules to objectively classify the aforementioned set of subjectively graded visual smoothness panels. Section 2.1 includes an overview of the standardized visual smoothness tiles investigated in this study. Section 2.2 includes acquisition and data-collection details of the tiles using a CSI and a gloss meter. Gloss meter readings are included in the analysis due to their simplicity. Areal parameters considered and spatial bandwidths used in generating the datasets for subsequent analysis are covered in Section 2.3. The parameter selection and classification methodology employed for down-selecting metrics and classifying the standard appearance tiles is covered in Section 3. The performance of a decision tree classifier and a summary of the down-selected parameters using this algorithmic methodology is covered in Section 4. A summary of the results and areas for future work is outlined in Section 5.

## 2. Samples and Measurements

### 2.1. Visual Smoothness Standards

**Figure 1:**
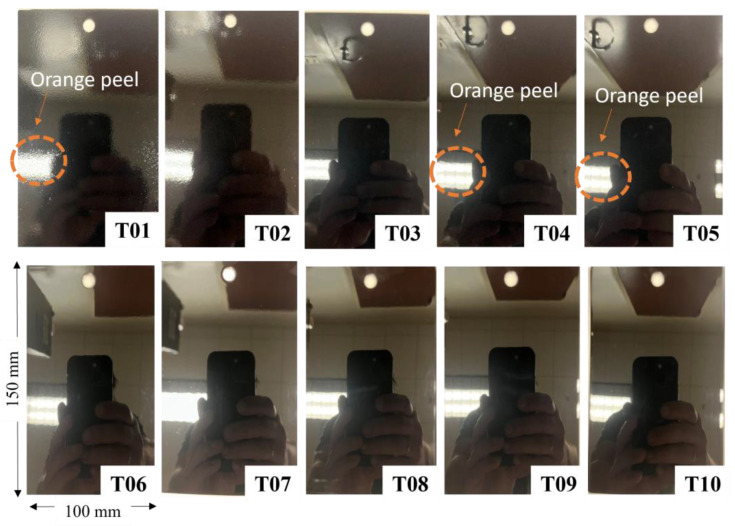
Images of PCI visual smoothness standards captured by an iPhone under non-ideal lighting conditions. Circular regions shown in T01, T04, and T05 illustrate the reflected image distortion caused by the severity of the orange peel.

The PCI visual smoothness standards shown in Figure 1 cost approximately 650 USD and consist of ten 100 mm × 150 mm black powder-coated tiles with varying degrees of smoothness. The samples are numbered such that tile 01 appears to be the “roughest” sample, containing the most orange peel, and tile 10 is the “smoothest”, visually exhibiting the least orange peel and the highest reflectance levels. The orange peel texture appears as its name suggests: visible local surface undulations or waviness. It is most visible in tiles 01 and 02 and within the region of the reflected overhead light in tiles 04 and 05 (see the indicated regions in Figure 1). Industrial equipment manufacturers prescribe different quality designations to surfaces with an orange peel rating of 05–09 versus those with a rating of 02–09 [37]. Typically, classification is carried out via HVI in illuminated cells by comparing the standard tile grades to the part surfaces. Under non-ideal lighting conditions, only PCI tiles 01, 02, and potentially tile 03 can be reliably differentiated. Resolving the differences between tiles 04 to 10 is more challenging, requiring specific lighting conditions, viewing angle, and visual acuity to resolve minor variations in spatial periods and specular reflection. To correctly classify all ten tiles over long periods of time would be considered a difficult inspection task and would undoubtedly lead to inconsistency between inspectors.

The PCI tiles in Figure 1 were kept in a protective case during shipping and storage. Direct handling was limited to the edges of each tile to avoid damage or contamination of the measurement area. Before each measurement described in the next section, the tiles were gently wiped with a clean microfiber cloth to remove any dust or contaminates on the surface. 

### 2.2. Gloss Meter and CSI Measurements

**Figure 2:**
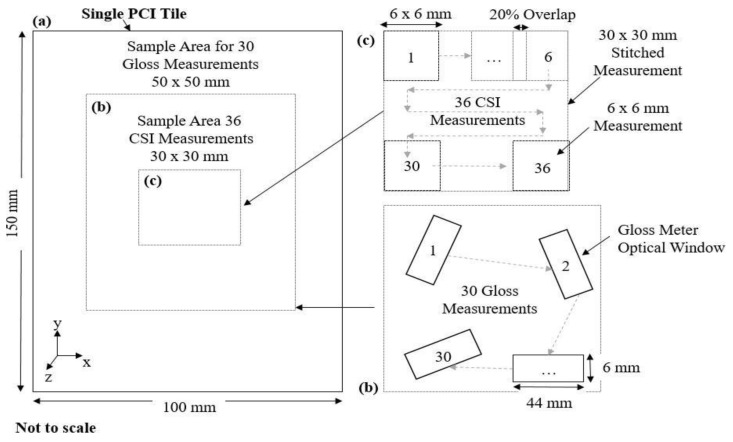
(**a**) Illustration of sampling locations on the PCI tiles for gloss meter (**b**) and CSI measurements (**c**).

The different measurement strategies for the two different measurement approaches are depicted in Figure 2. Thirty measurements of each PCI tile were collected using a RhoPont-IQ meter [29]. This was achieved by rotating about the z-axis normal to the surface and translating the device across the central region of the 100 mm × 150 mm tiles. The assessment areas were uniformly distributed over approximately 50 mm × 50 mm center region of the tile, maintaining a buffer around the edges to prevent inaccurate readings. The PCI tiles would generally be considered “high-gloss” surfaces; as such, 20° angle gloss measurements are most appropriate for quantifying the tiles compared to 85° and 60° angles. By design, *DOI*, *R_spec_*, and *RIQ* assessments are expected to correlate with the severity of orange peel, which is assumed to determine the ascending ordering of the tiles, i.e., from tile 01 to 10. Surface measurements of the PCI visual smoothness standards were obtained using a Zygo NexView CSI (Middlefield, CT, USA) configured with a Michelson 2.75× objective and 0.5× tube lens, providing a numerical aperture (NA) of 0.08 and sampling interval of 5.91 µm. The objective chart for the instrument uses the term spatial sampling in units of µm per pixel to describe the latter, noting it is the pixel size on the sample and is derived from the camera pixel size divided by the system magnification. The instrument is housed in a laboratory environment temperature controlled to 20 ± 0.1 degrees C. Plastic toe clamps were used to secure and flatten each tile to the stage of the CSI. The CSI measurements were taken from the center region of the tile. The measurements comprise partially overlapping 6.05 mm × 6.05 mm scans stitched together to increase the nominal field of view to 30.25 mm × 30.25 mm. Stitching enables a larger field of view without comprising the measurement’s spatial resolution. The stitching process was carried out using Zygo MX™ (v8.0.0) software using 20% overlap between sequential scans in the adaptive adjust mode. 

#### 2.2.1. Measured Gloss Metric Values

**Figure 3:**
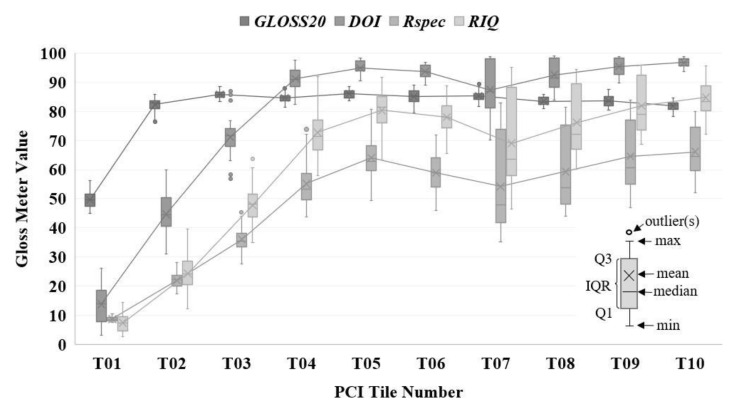
Box and whisker plots of select Rho-Point IQ gloss meter readings vs. PCI tile number.

Figure 3 depicts box and whisker plots highlighting the relationship between the values of selected gloss metrics (*y*-axis) and PCI tile number (*x*-axis). Gray boxes represent the interquartile (IQR) range, with whiskers denoting the minimum and maximum quartiles. Each box displays the median and mean of the data. The median is indicated by a horizontal line, while the mean is represented by “×” symbol(s). Outliers are shown as circles.

For *Gloss*20, there is a clear distinction between tile 01 and tile 02, with average values of approximately 50 and 83 gloss units, respectively. Metrics *DOI*, *R_spec_*, and *RIQ* demonstrate a positive correlation for tile 01 through tile 04 (the mean value of the measurements increases as a function of the tile number.). However, even using the data illustrated in Figure 3, it is clear that no one of the functional parameters (*Gloss*20, *DOI*, *R_spec_*, *RIQ*, etc.) would enable the ability to distinguish between all ten tiles, nor facilitate the ability of a machine learning model to automate the classification of new tiles. This is because there is too much overlap between metric values computed on tile 04 through tile 10. For example, the range of *Gloss*20 values for T04, T05, T06, and T07 are almost identical.

#### 2.2.2. Measured CSI Data and the Power Spectral Density Curves

**Figure 4:**
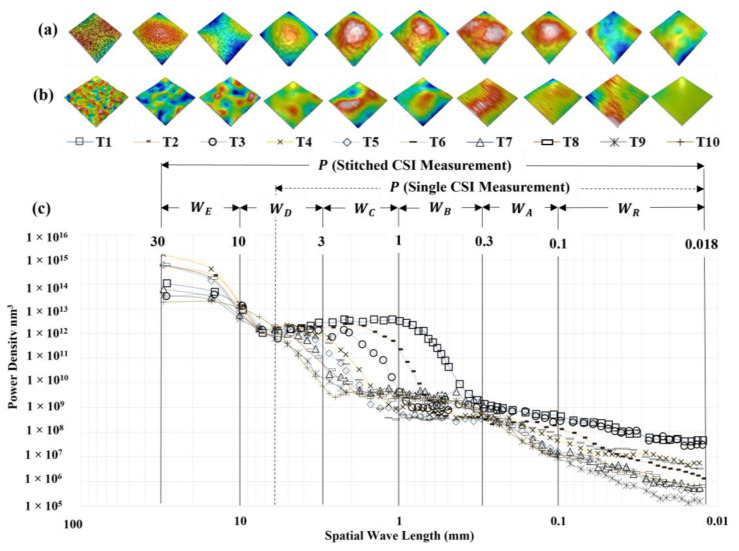
(**a**) Examples of stitched 30.25 mm × 30.25 mm CSI measurements. (**b**) Examples of single 6.05 mm × 6.05 mm CSI measurements. (**c**) Power spectrum density curves of stitched 30.25 × 30.25 mm CSI measurements of PCI tiles 01–10 with spatial bands *W_E_*, *W_D_*, *W_C_*, *W_B_*, *W_A_*, and *W_R_* overlaid.

The images at the top of Figure 4a show stitched 30.25 mm × 30.25 mm CSI measurements, with typical 6 mm × 6 mm single CSI measurements taken from each tile shown below (Figure 4b). The lower section, Figure 4c, depicts the Power Spectral Density (PSD) for each of the ten stitched measurements (30.25 mm × 30.25 mm). The PSD of a surface is the average of the square of the Fourier transform for each row (or column) of an areal measurement [38]. The graph provides insights into the spatial content of a surface, whereby the *x*-axis covers the range of spatial wavelengths possible to capture within a measurement. In Figure 4, a non-directional PSD was calculated using Zygo MX™ (v8.0.0) for each 30.25 mm × 30.25 mm stitched CSI measurement (see Figure 2c) using default settings. The non-directional PSD is calculated by summing the 2D PSD data for a single frequency in all directions for each frequency for which there is data. Readers are referred to the MX™ (v8.0.0) software manual for complete details. In this case, the highest frequency coincides with the sampling interval of 5.9 µm, limited by the pixel size of the CSI detector and system magnification. The lowest frequency is bounded by the 30.25 mm dimension(s) of the stitched measurement. However, the longest feasible wavelength discernible with any degree of confidence is approximately 7 mm, i.e., one-quarter of the overall length of the stitched measurement. This is slightly larger than the nominal field-of-view of the single 6 × 6 mm CSI measurements (see Figure 2c).

Different surface features (micro-scale surface roughness affecting reflectance or longer-scale surface undulations referred to as orange peel) occupy different length scales on the surface; both influence the classification of the ten different tiles. However, exactly which surface features and length scales correlate with the visual ranking of the tiles is unknown. Therefore, the surface is split into five different spatial bands for further analysis. The bands (cut-off-frequencies) chosen are those commonly used to access paint finish in the automotive industry [39,40,41]: *W_A_* (0.1 mm → 0.3 mm), *W_B_* (0.3 mm → 1 mm), *W_C_* (1 mm → 3 mm), *W_D_* (3 mm → 10 mm), and *W_E_* (10 mm → 30 mm). The additional band *W_R_* (0.018 mm → 0.1 mm) is included to account for the shortest spatial content on the surface, i.e., between three times the sampling interval and the lowest 0.1 mm cutoff of the *W_A_* band. The additional P bands indicated the unprocessed surface data of either the larger stitched file P_Stitched_ or a single measurement from the stitch, P_single_.

Integrating the PSD curve(s) shown in Figure 4 across the entire range of spatial frequencies would result in the total power of the primary surface(s) comparable to the squared root-mean-square roughness (*Sq*) of the stitched measurement(s). Band-limited calculations of *Sq* can be obtained using the stated cut-offs of a given band as the bounds for integration. The *W_D_* band shows a more “ideal” (strictly negative) correlation between amplitude parameters and tile number (relative to other bands); T01 has the highest band-limited *Sq* value (0.37 µm), and *Sq* generally decreases with every other subsequent tile number. However, the *Sq* value for T10 (0.124 µm) is approximately equal to that for T09 (0.123 µm), the *Sq* value of T04 (0.276 µm) is greater than T03 (0.261 µm), similar for T06 (0.243 µm) and T05 (0.230 µm). *W_D_* does not provide the required amount of separation that is needed for reliable classification. Using cut-offs for the *W_A_* or *W_R_* band would result in the *Sq* of T03 greater than T02. Bands *W_B_* and *W_C_* would rank T09 and T10 above T04, T05, and T06. Band *W_E_* does not rank the sample correctly, either. For subsequent analysis, only *W_A_*, *W_B_*, and *W_C_* bands are used on the 360 individual smaller measurements (6.05 mm × 6.05 mm). The additional band *W_R_* that captures shorter wavelengths (0.1 mm → 0.018 mm) and a P band (the primary surface) will also be used. Limiting the evaluation to these bandwidths eliminates the need to obtain stitched measurements to accommodate the field of view (FOV) required for bands *W_E_*, *W_D_*, which in turn reduces overhead in data storage (24 MB vs. 262 MB), measurement time (~4 s vs. 10 min), and subsequent processing time (~1 s vs. 10 s).

### 2.3. Dataset Generation

Table 1 summarizes the acquisition details, measurements, and parameters used in the subsequent analysis. The field of view (FOV) for Rhopoint-IQ measurements varies depending on the calculated metric. For the 85° gloss metric, it corresponds to 4.4 mm × 44 mm; for the 60° gloss, a 6 mm × 12 mm area is required; and for the 20° gloss, *DOI*, *RIQ*, and *R_spec_* 6 mm × 6.4 mm definition areas are needed. The gloss dataset consists of only these eight parameters provided by the Rhopoint-IQ. Twenty-one ISO 25178-2 parameters were computed for each CSI measurement after the pre-processing operations listed below in Table 2 were applied. The specified filtering operations and subsequent parameter calculations were performed using Zygo MX™ (v8.0.0) software [42]. Each unique dataset, *P*, *W_C_*, *W_B_*, *W_A_*, and *W_R_*, consists of 360 individual measurement sites (6.05 mm × 6.05 mm), 36 per sample, and 22 ISO parameters calculated for each of the 360 areal maps. The tabulated results were exported in .csv format for subsequent analysis. For brevity, only the shorthand abbreviations of the ISO 25178-2 areal parameters are included in Table 1; readers are referred to the standard itself [32] or to references [19,43] for detailed verbal descriptions and numerical definitions of each parameter. 

**Figure 5:**
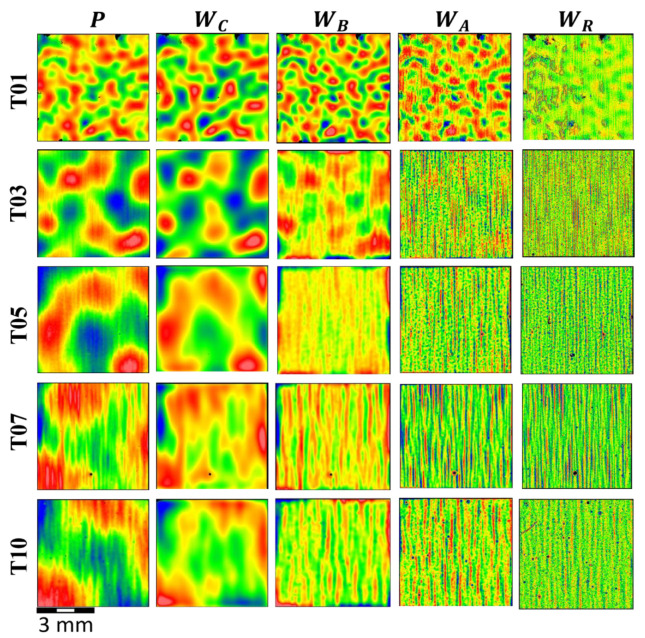
Examples of CSI measurements after pre-processing operations used to create datasets *P*, *W_C_*, *W_B_*, *W_A_*, and *W_R_*. Color ranges have been optimized for each image to improve viewing quality. Red and blue colors correspond to areas of higher or lower elevation, respectfully.

Table 2 includes the processing information for each dataset, specifying the low-pass filter, bandpass filter, form removal, spike removal, and edge clipping applied to data prior to computing ISO 25178-2 parameters. The cut-off wavelength of the initial lowpass Gaussian spline filter used in each case is set to three times the sampling interval to remove high-frequency noise from the data. Note that the sampling interval remains constant, irrespective of the filtering applied to the data. A few false height readings were observed in the measurements, likely caused by dust particles or contamination on the surface. Although these will have little effect on the values of texture parameters such as the average roughness (*Sa*) or root mean square gradient (*Sdq*), as the million other pixel values will average them out, a single outlier in the measurement can significantly affect the extreme value parameters such as the maximum peak height (*Sp*), maximum pit height (*Sv*), and maximum height (*Sz*); therefore, spike clipping is used to ensure these parameters characterize the actual topography and not single-pixel measurement artifacts. The spike filter is set at 6σ; the maximum number of data points removed from any measurement was less than 0.3% and visually has no impact on the topography image or values of other ISO parameters. Edge cropping was employed to remove filtering artifacts. Examples of processed height maps after bandpass filtering are shown in Figure 5.

Optimized color representation for each height map shown in Figure 5 makes it much easier to see differences in the spatial aspects of the topography than if a fixed color bar (height scale) was used for the image or any row/column; as such, the colors depict different height ranges for each image. The primary surface (*P*) of tile 01 exhibits an isotropic topography (no apparent surface structure or directionality). However, traveling down the same column from T01 to T10, notice that as the degree of isotropic orange peel (longer undulations) decreases, there is an increase in anisotropic vertical striations. Still, identifying unique surface characteristics that distinguish between tiles 05, 07, and 10 is extremely difficult, regardless of the applied filtering.

**Figure 6:**
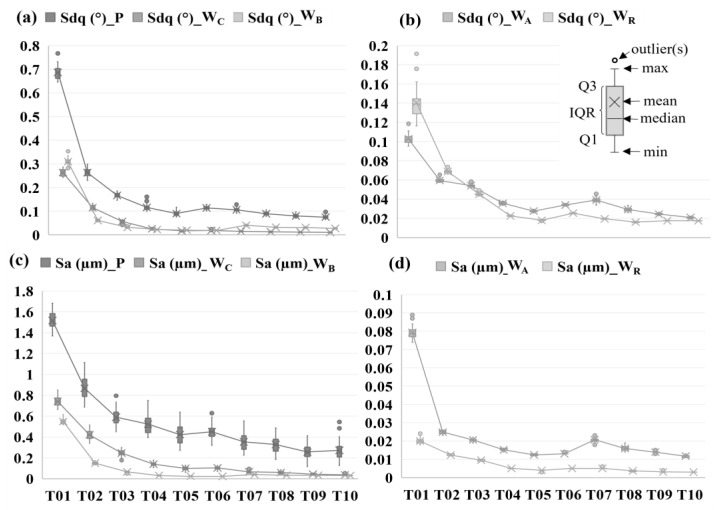
(**a**) *Sdq* (*P*, *W_C_*, *W_B_*) vs. tile number. (**b**) *Sdq* (*W_A_*, *W_R_*) vs. tile number. (**c**) *Sa* (*P*, *W_C_*, *W_B_*) vs. tile number. (**d**) *Sa* (*W_A_*, *W_R_*) vs. tile number.

Figure 6 depicts the areal roughness (*Sa*) and the root-mean-square-gradient (*Sdq*) of the tile surfaces at the different spatial bands. Notice the lack of a strictly negative correlation between parameter values and the tile number. For the primary surface (the *P* dataset shown in Figure 5), the areal surface roughness *Sa* of the 10 tiles ranged from 0.25 µm to 2 µm, *Sq* from 0.15 µm to 2.02 µm, and *Sdq* ranged from 0.07° to 0.77°. A fourth-order fit between *Sdq* and tile number yields correlation coefficient *R*^2^ values of 0.98, 0.99, 0.96, and 0.96 for *P*, *W_C_*, *W_B_*, and *W_A_* datasets, respectively. Similar *R*^2^ values were observed for *Sa*. Although there is a high correlation, the fitted function will not enable explicit classification criteria; there is insufficient separation in the metrics’ values for the higher-numbered tiles. While not shown here, none of the other 20 ISO 25178-2 parameters considered delivered clear distinctions and/or strictly linear trends between all ten tiles.

## 3. Parameter Selection and Classification Methodology

*Surface Quality and Inspection Descriptors (SQuID* *™)*

Instead of relying on preconceived notions of parameter selection for accurately classifying the PCI tiles, an algorithmic method outlined in [21] called *SQuID*™ (Surface Quality and Inspection Descriptors) is used to down-select optimal surface parameters for classification. *SQuID*™ uses a selection method that breaks down a user-defined multi-class classification task into a series of binary classification tasks. For instance, in this case, the multi-class task involves using areal texture or gloss descriptions from Nexview CSI or Rhopoint-IQ to determine a measured tile designation (01, 02, 03 … 10). The procedure begins by decomposing the multiclass task into a finite series of binary classification tasks (T01 vs. T02, T01 vs. T03, and so on). For each binary task, a reference set of measurements quantified by a single areal or gloss description is sampled from the dataset. The difference in means between the two groups is divided by the root-mean-square of the standard deviations. Taking the absolute value of this resulting quantity yields a discriminability index referred to as *d*′, describing the separation between the two groups in terms of normalized standard deviations. The process is repeated for each parameter on each binary classification task derived from the multi-class dataset, resulting in a *d*′ matrix. In this matrix, parameters are indexed as rows, and binary classification tasks are represented as columns. The optimal set of parameters for the multi-class task is derived by selecting the parameter (row index) with the maximum *d*′ value for each task (column index). If, for every binary task, the corresponding selected parameter exhibits a *d*′ value of approximately seven or higher, a reasonably optimized decision tree classifier trained using these selected parameters is expected to achieve near-perfect classification accuracy on the multi-class dataset. Following the down-selection process, *SQuID*™ employs a decision tree as the default machine-learning model to determine a logical mapping between the selected parameters and the target surface classes. The fitted decision tree can intuitively communicate how the selected surface texture parameters and associated thresholds can be employed as rules for classifying a newly measured surface, providing an objective basis for assessing surface quality in accordance with user-defined grades of surface quality. Readers are referred to the original paper [21] for complete details, including examples of the selection procedure, construction of the *d*′ matrix, and graphs related to the expected classification outcomes versus *d*′ values of the selected parameters.

## 4. Results

### 4.1. Summary of d′ Matrix

**Figure 7:**
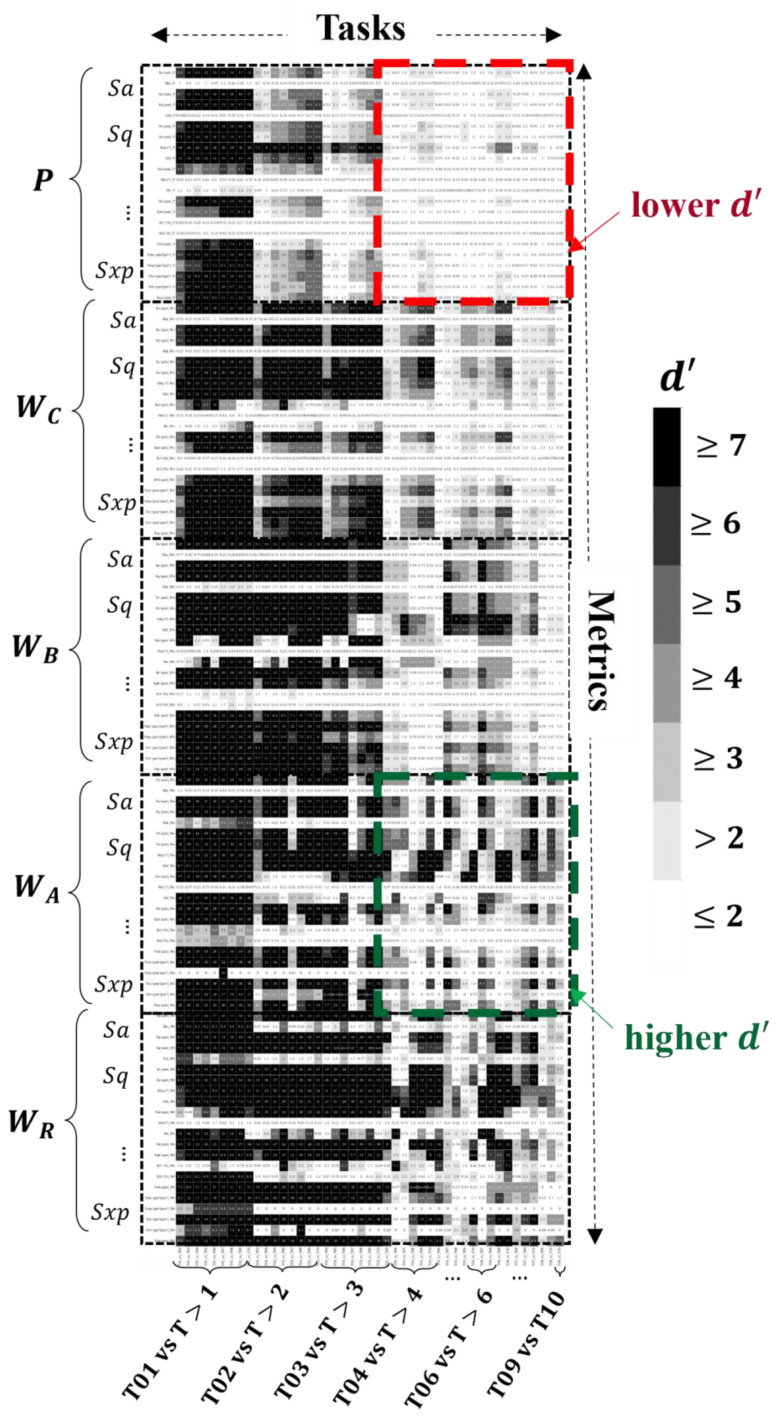
Example of the *d*′ matrix generated for combined datasets. *P*, *W_C_*, *W_B_*, *W_A_*, and *W_R_* comprise 110 metrics (rows) and 45 tasks (columns).

Figure 7 represents the combined *d′* matrix generated for the *P*, *W_C_*, *W_B_*, *W_A_*, and *W_R_* dataset(s). Columns are indexed as tasks following the sequence T01 vs. T02, T01 vs. T03, etc., and rows are indexed as ISO parameters for each band (see Table 1 for ordering). For this discussion, reading the fine print associated with the rows and columns on the matrix is not important; what is important is the distribution of the gray values on the *d*′ matrix. The shading represents the magnitude of the *d*′ values computed for each row (parameter)–column (task) combination. Ideally, one row (metric) would have a *d*′ value greater than seven (black shading) across all columns (tasks), though this is not the case for any datasets considered. Notice that the *d*′ values for most ISO parameters along the first column (task T01 vs. T02) tend to be greater than those of the last column (task T09 vs. T10). This implies that differentiating between the topography of tiles 01 and 02 using ISO parameter values is relatively trivial compared to 09 and 10, regardless of the pre-processing routine applied to the data. The *d*′ matrix also suggests that a classification model that uses metrics computed for the *P* and *W_C_* bands will perform worse than a model that uses ISO metrics computed for the *W_B_* and *W_A_* bands. This is because there is an increased coverage of higher *d*′ values across the entire task space in the *W_A_* and *W_B_* bands. A combination of metrics with dark values (*d*′ ≥ 7) across all tasks implies the data are likely separable, and near-perfect classification performance is expected. The expanse of white cells (*d*′ < 2) in the *P* and *W_C_* bands implies less-than-ideal performance (≈80% classification accuracy or less); see the experimental curve in [21] for the empirical relationship between *d*′ and classification accuracy. The relevant regions are highlighted by the dashed red and green boxes in Figure 7.

### 4.2. Identified Parameters and Classification Performance

Table 3 indicates the top metrics identified by *SQuID*™ for each analysis set and the classification performance of a fitted decision tree that used the selected metrics to predict the tile number (i.e., surface grade). The average and standard deviation of the test accuracy based on stratified five-fold shuffle split cross-validation [44] using train/test splits of 0.1/0.9, 0.5/0.5, and 0.9/0.1 for each dataset is shown in the rightmost columns. The evaluation provides a more comprehensive estimate of the expected classification performance compared to using a single dataset for training and testing. The first row of Table 3 indicates that gloss metrics fail to provide enough information to discern between the ten standard surface tiles. This comes as no surprise given the overlap of the gloss data illustrated in Figure 3; no reduction in the number of selected features is observed, and average accuracies as low as 58% are recorded for the 0.1/0.9 split, 74% for the 0.5/0.5 split, and 75% for the 0.9/0.1 split. For the primary dataset *P*, a total of five features are selected by *SQuID*™. Still, they fail to perform any better than the gloss metrics, averaging around 65% classification accuracy or less across all train/test splits. This is unsurprising given that only 2% of metrics had values of *d*′ > 3 for tasks T04 vs. T > 04 (see the dashed red box in the top right corner of Figure 7). Coincidentally, this is where most incorrect classifications occurred. Classification accuracy marginally improves between 5% and 8% depending on the train/test split for the *W_C_* dataset, where seven features are selected. Again, better performance is expected; the *d*′ values in Figure 7 for this dataset tend to be greater than those of the ISO metrics of the *P* dataset. For the latter, only 31% of all entries had *d*′ values greater than three, with the former having 50%. For tasks T04 vs. T05 to T10, 29% of entries were greater than three. Most incorrect classifications occur between T08, T09, and T10. Seven metrics (with higher *d*′ values) were also selected for the *W_B_* dataset; the classification performance ranges between 78% to 82%, approximately a 10% increase over the *W_C_* dataset. However, again, most incorrect classifications occur for tasks T08 vs. T09 and T10, where none of the *d*′ values of the selected metrics were greater than four. A drastic increase in performance is observed for the *W_A_* and *W_R_* datasets. The former achieved an average accuracy of 97% using ten selected features, and the latter reached 98% based on eleven selected parameters for the 0.5/0.5 split. Both consist of selected metrics with *d*′ > 4 for tasks T08 vs. T09 and T10. Less than five incorrect classifications in total were recorded in both cases. For the combined dataset (i.e., *P*, *W_C_*, *W_B_*, *W_A_*), which includes 88 ISO features, near-perfect classification is achieved for the 0.5/0.5 and 0.9/0.1 splits using only five multi-scale metrics selected from the *W_B_* and *W_A_* datasets. Less than five incorrect predictions were made, each being off by a single tile designation and occurring only on tasks T07 vs. T08 to T10. The fact no ISO metrics were selected from the *P* and *W_C_* datasets indicates there was always an ISO metric computed on either the *W_B_* or *W_A_* dataset that possessed a greater *d*′ value for a given classification task (column(s) on the *d*′ matrix).

### 4.3. Interpretation of Classification Criteria

**Figure 8:**
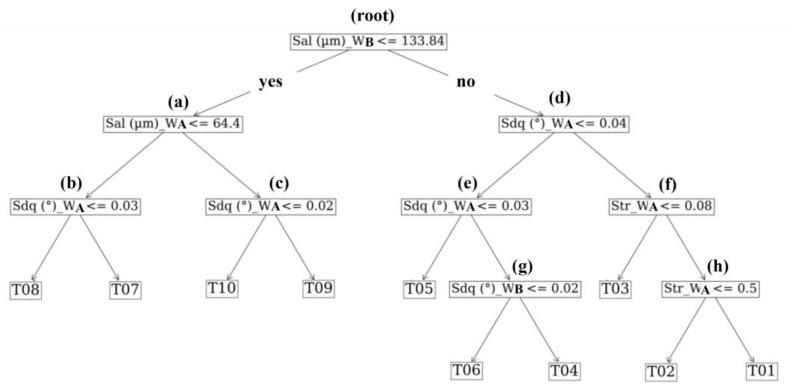
Decision tree generated from *SQuID*™ based on five parameters selected from combined *P* + *W_C_* + *W_B_* + *W_A_* datasets. For visualization, parameter values are truncated to two decimal places. Callouts (**a**–**h**) represent the logic used to classify a surface as one of the ten tile grades.

**Figure 9:**
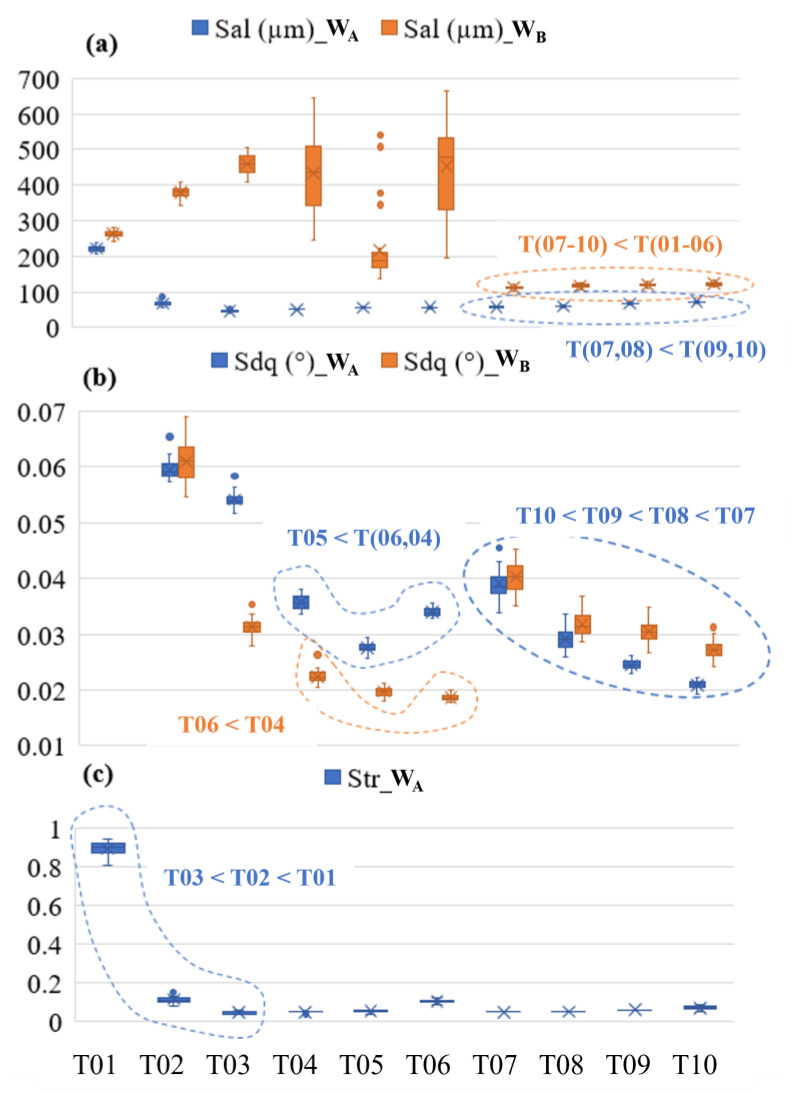
Decision tree verification by graphical assessment of the parameters selected by *SQuID*™ for classification. (**a**) Measured auto-correlation length *Sal* and (**b**) rms-slope *Sdq* values for *W_A_* and *W_B_* bands plotted against tile designation T01–T10. (**c**) Texture aspect ratio *Str* values computed for the *W_A_* band of the ten different tiles.

Figure 8 depicts the decision tree generated using down-selected parameters, including the auto-correlation length *Sal* and root-mean-square gradient *Sdq* computed for the *W_B_* and *W_A_* bands, in addition to the texture aspect ratio *Str* computed on the *W_A_* band. Figure 9 plots the values of the ISO metrics selected for use in the decision tree for all ten tiles. Examination of the three graphs illustrates that a combination of metrics can exist to differentiate the tiles from each other but that no one metric could differentiate between all ten; the decision tree is required to achieve the latter. Verification of the decision tree logic used for classifying the data is depicted in Figure 9. Following the logic of the tree (Figure 8), the first split for differentiating tiles 01–06 from 07–10 is by comparing autocorrelation length *Sal* on the *W_B_* surface to a threshold of 133 µm (the root node top of the tree). Based on inspection of the data in Figure 9a, it is obvious that if the *Sal_W__B_* value of the surface in question is less than or equal to this value, a classification of grades 07–10 is most appropriate. The next split to the left of the root node (decision node a) compares the *Sal* of the surface to a value of 64.4 µm but, in this case, on the *W_A_* surface, differentiating a tile 07 or 08 from a 09 or 10 designation. Finally, a minor difference in the *Sdq* of the surface for spatial band *W_A_* is used to assign a final designation of 07, 08, 09, or 10 (see decision nodes b and c). Following the logic to the right of the root node (decision node d), the *Sdq* of the *W_A_* band is used to split the group of designations (04, 05, 06) from (01, 02, 03). In the case of the latter group, the presence of what appears to be rolling marks on the surface, exhibiting a dominant lay pattern, becomes more noticeable as the degree of orange peel is reduced, resulting in a departure from an isotropic texture (see Figure 5). This trend in increasing anisotropy is captured by the texture aspect ratio (*Str*) at the *W_A_* band shown at the bottom of Figure 9c, which can be used to resolve the individual differences. For the former group (04, 05, 06), it is important to note that the *Sdq* for the *W_B_* band is less for tile 05 than for 04 and 06. This initial split (decision node e), followed by comparing the *Sdq* value for the *W_B_* band, enables individual designations (see Figure 9b and decision node g in Figure 8).

This result highlights a unique approach for characterizing topography with ISO 25178-2 parameters, which does not limit the evaluation to a single pre-processing routine. Using a script configured to run the CSI and automate the data processing/evaluation steps would enable the classification of new surfaces in less than 10 s, bypassing visual inspection. The parameter values in Figure 9 and decision tree logic in Figure 8 are subject to change for different measurement conditions and data processing. For example, the calculated values of hybrid parameters *Sdq* and the interfacial area ratio *Sdr* depend on the sampling interval. This can be modified by adjusting the system magnification of the CSI or increased digitally after the measurement is acquired. The use of different filter types, instrument settings, and environmental disturbances such as temperature and vibrations can also affect parameter values. Further study is required to evaluate the impact of these conditions on classification performance in addition to different modes of acquisition, such as confocal and profilometry.

## 5. Summary and Conclusions

This work demonstrates the ability of a multi-scale methodology called *SQuID*™ to down-select both ISO 25178-2 and gloss metrics to quantify ten different grades of powder-coated surfaces objectively. A set of only five multi-scale quantitative descriptions of the surface coupled with interpretable decision logic was provided to classify ten PCI visual smoothness standard tiles with near-perfect classification accuracy. This performance was achieved despite a limited sample size and nonlinear correlation between the qualitative ranking of the tiles (grades 01–10) and the values of parameters obtained from CSI measurements of each sample. Moreover, in cases where incorrect classification occurred, the predictions were only off by a single sample grade. Central to achieving this is leveraging the fact that a tile’s texture comprises features of varying spatial wavelength and amplitude; this is apparent from the varied PSD trends for each tile illustrated in Figure 4c and the bandwidth-filtered measurements depicted in Figure 5 and Figure 6. While features of different wavelength variations may not always be resolvable by a human’s eye (if the amplitude is too low or the wavelength is too short), they all affect a light’s interaction with a surface and, hence, how the same is perceived. Filtering at selected bandwidths enables their isolation, and subsequent quantification provides the additional metrics necessary for successful correct classification. For this work, filtering bandwidths used in the automotive industry for powder-coating and painting processes were utilized. No effort was expended on isolating optimal bandwidths; however, pursuing this avenue could yield even better outcomes. Incorporating additional segmentation methods and feature parameters defined within the ISO 25178-2 standard may also yield even more robust and accurate classification. Of equal importance is to note that the presented classification criteria(s) provide the explicit logic to make predictions; it is not a black box function. It is possible to use this decision rule diagram in an automated way with a machine vision or measurement system and/or as a reference to support visual inspection effects when subjectivity arises. Overall, this approach demonstrates great potential in removing the need for visual inspection. In conclusion, the *SQuID*™ framework is shown to be a practical, systematic, interpretable, multi-scale approach for selecting standardized statistical parameters to transform subjective grades of surface quality into objective descriptions of surface topography. 

## Figures and Tables

**Table 1:** Instrument specifications and datasets collected for analysis.

Instrument	Zygo NexView	Rho-Point IQ
Objective	2.75×	n/a
Tube Lens	0.5×	n/a
Field of View (FOV)	6.05 mm × 6.05 mm	6.04 mm × 6 mm 6 mm × 12 mm 4 mm × 44 mm
Sampling Interval	5.9 µm	n/a
Sampling Area	30.25 mm × 30.25 mm	50.0 mm × 50.0 mm
Number of Measurements	36/Sample	30/Sample
Metrics	*Sa*, *Sq*, *Ssk*, *Sku*, *Sp*, *Sv*, *Sz*, *Sdq*, *Sdr*, *Sal*, *Str*, *Std, Sk*, *Spk*, *Smr*1, *Smr*2, *Svk*, *Vmc*, *Vmp*, *Vvc*, *Vvv*, *Sxp*	*Gloss*20, *Gloss*60, *Gloss*85, *Haze*, *LogHaze*, *DOI*, *RIQ*, *R_spec_*
References	[32,42]	[27,29]

Note: n/a refers to either not available or not applicable.

**Table 2:** Detailed information on datasets and filtering operations: low-pass, bandpass, form removal, spike removal, and edge clipping.

Dataset	Low-Pass	Bandpass	Form Removal	Spike Removal	Edge Clipping
*P*	18.0 µm	-	Best fit plane	6σ from mean plane	No
*W_C_*	18.0 µm	1.0 mm–3.0 mm	Best fit plane	6σ from mean plane	24 pixels
*W_B_*	18.0 µm	0.3 mm–1.0 mm	Best fit plane	6σ from mean plane	24 pixels
*W_A_*	18.0 µm	0.1 mm–0.3 mm	Best fit plane	6σ from mean plane	24 pixels
*W_R_*	18.0 µm	0.018 mm–0.1 mm	Best fit plane	6σ from mean plane	24 pixels

**Table 3:** Classification accuracy of decision tree based on selected features for seven different datasets.

Dataset	# Input Features	Selected Features	Test Accuracy * Stratified 5-Fold Shuffle Split		
Gloss	8	*Gloss*20, *Gloss*60, *Gloss*85, *Haze*, *LogHaze*, *DOI*, *RIQ*, *Rspec*	0.58 ± 0.045	0.74 ± 0.013	0.75 ± 0.063
*P*	22	*Sv*, *Sp,* *Sdq*, *Std*, *Sz*	0.56± 0.04	0.65 ± 0.025	0.65 ± 0.075
*W_C_*	22	*Sz*, *Vmc*, *Sa*, *Vvc*, *Sv*, *Sq*, *Sdq*	0.61 ± 0.063	0.68 ± 0.013	0.73 ± 0.025
*W_B_*	22	*Sp*, *Vmc*, *Sdq*, *Sal*, *Sdr*, *Str*, *Sz*, *Sk*	0.78 ± 0.02	0.81 ± 0.02	0.82 ± 0.02
*W_A_*	22	*Sz*, *Vvv*, *Vmp,* *Sp*, *Sal*, *Sa*, *Vvc*, *Sv*, *Sdq*, *Sdr*	0.91 ± 0.04	0.97 ± 0.01	1.00 ± 0.00
*W_R_*	22	*Spk*, *Sdq*, *Sdr*, *Sal*, *Vvv*, *Sxp*, *Str*, *Svk*, *Vvc*, *Sk*, *Vmp*	0.91 ± 0.02	0.98 ± 0.02	0.99 ± 0.01
*P+W_C_ + W_B_* + *W_A_ +W_R_*	88	*Sdq_W__B_*, *Sal_W__B_*, *Str_W__A_*, *Sdq_W__A_*, *Sal_W__A_*	0.91 ± 0.02	0.99 ± 0.01	0.99 ± 0.01
Training/Testing		Split:	0.1/0.9	0.5/0.5	0.9/0.1
		** Number Samples:	40/320	180/180	320/40
		** Number Samples per Tile:	4/32	18/18	32/4

* Accuracy is computed as the number of correct predictions divided by the total number of predictions made on the test set. ** The number of samples and samples per class is different for the gloss dataset. # Refers to the number of input features to *SQuID*™.

## Data Availability

Data are contained within the article.
